# Vitamin D deficiency in relation to the poor functional outcomes in nondiabetic patients with ischemic stroke

**DOI:** 10.1042/BSR20171509

**Published:** 2018-03-05

**Authors:** Zhao-Nan Wei, Jian-Guo Kuang

**Affiliations:** 1 The First Clinical Medical College, Nanchang University, Nanchang, China; 2 Department of Neurosurgery, First Affiliated Hospital of Nanchang University, Nanchang, China

**Keywords:** 25-hydroxyvitamin D, functional outcome, ischemic stroke, mortality, nondiabetic

## Abstract

To assess the hypothesis that vitamin D, reflected by 25-hydroxyvitamin D (25(OH) D) would be associated with higher risk of poor functional outcomes amongst nondiabetic stroke patients. The present study was conducted in Nanchang, China. Serum concentration of 25(OH) D and National Institutes of Health Stroke Scale (NIHSS) were measured at the time of admission. Functional outcome was measured by modified Rankin scale (mRS) at 1 year after admission. Multivariate analyses were performed using logistic regression models. The cut point of 25(OH) D level for vitamin D deficiency was 20 ng/ml. In the present study, 266 nondiabetic subjects with stroke were included; 149 out of the 266 patients were defined as vitamin D deficiency (56%). The poor outcome distribution across the 25(OH) D quartiles ranged between 64% (first quartile) and 13% (fourth quartile). In those 149 patients with vitamin D deficiency, 75 patients were defined as poor functional outcomes, giving a prevalence rate of 50% (95% confidence interval (CI): 42–58%). In multivariate analysis models, for vitamin D deficiency, the adjusted risk of poor functional outcomes and mortality increased by 220% (odds ratio (OR): 3.2; 95% CI: 1.7–4.2, *P*<0.001) and 290% (OR: 3.9; 95% CI: 2.1–5.8, *P*<0.001), respectively. Vitamin D deficiency is associated with an increased risk of poor functional outcome events in Chinese nondiabetic stroke individuals.

## Introduction

In China, the annual stroke mortality rate is approximately 1.6 million, which has exceeded heart disease to become the leading cause of death and adult disability [1]. Early and accurate prediction of outcomes in stroke is important and influences risk-optimized therapeutic strategies.

Vitamin D, which is purely considered as a hormone that primarily regulates calcium metabolism, displays a strong anti-inflammatory role in the current researches [2]. Vitamin D deficiency (defined as 25-hydroxyvitamin D (25(OH) D) <20 ng/ml) has been proposed as a new risk factor for cardiovascular disease (CVD) [3–5], including stroke [6] and diabetes [7]. Some prospective studies indicated that lower 25(OH) D concentration was associated with a higher risk of poor functional outcomes and all-cause mortality amongst ischemic stroke patients at different time points [8–10].

Hyperglycemia is common in patients with acute stroke attributed to stress response or previous diabetes mellitus [11]. Considering the close relationship between 25(OH) D and blood glucose [12,13], whether the effect of 25(OH) D on ischemic stroke prognosis is modified by blood glucose concentrations needs further elucidation. We hypothesized that vitamin D, reflected by 25(OH) D would be associated with higher risk of worse outcomes amongst nondiabetic stroke patients. We designed a prospective study to test this hypothesis in 266 Chinese nondiabetic patients with acute ischemic stroke.

## Methods

From June 2015 to May 2016, consecutive nondiabetic subjects with ischemic stroke admitted to the Department of Neurology of the First Affiliated Hospital of Nanchang University, China, were identified. Brain computer tomography (CT) or MRI and electrocardiography were performed in all patients. Specific additional inclusion criteria for the present study comprised (i) availability of blood samples, (ii) no diabetes before admission (diabetes at baseline was defined as use of or oral hypoglycemic drugs, glycated hemoglobin (HbA1c) level ≥6.5%, a fasting plasma glucose (FPG) ≥ 7.0 mmol/l, or a random serum glucose ≥11.1 mmol/l), and (iii) admission glycemia of <7.0 mol/l. In addition, patients with malignant tumor, head trauma, liver and kidney dysfunction, severe edema, and lost follow-up were also excluded. The present study was approved by the ethics committee of the First Affiliated Hospital of Nanchang University. All participants or their relatives were informed of the study protocol and their written informed consents were obtained.

Clinical information was collected. Demographic data (age and sex), body mass index (BMI), and history of risk factors (hypertension, hyperlipidemia, CVD, smoking habit, and alcohol abuse) were obtained at admission. Pre-stroke (oral anticoagulants, and statins) and acute treatment (IV thrombolysis and/or mechanical thrombectomy) were recorded. Clinical severity was assessed at admission using the National Institutes of Health Stroke Scale (NIHSS). If MRI was performed (*n*=148), the infarct volume was calculated using the following formula: 0.5 × a × b × c (where *a* is the maximal longitudinal diameter, *b* is the maximal transverse diameter perpendicular to *a*, and *c* is the number of 10-mm slices containing infarct). Functional impairment was evaluated at 1 year after admission using the modified Rankin scale (mRS). A good functional outcome of stroke patient was defined as a mRS score of 0–2 points, while poor functional outcome was in the range of 3–6 points [14]. Strokes were classified according to the criteria of the Trial of Org 10172 in Acute Stroke Treatment (TOAST) classification [15]. The clinical stroke syndrome was determined applying the criteria of the Oxfordshire Community Stroke Project (OCSP) [16].

Blood samples were drawn on the first morning (07:00) after admission under fasting state and within 48 h of onset of stroke symptoms/signs (within 0–6 h (*n*=49), 6–12 h (*n*=57), 12–24 h (*n*=84), and 24–48 h (*n*=76) from the symptom onset. Serum samples were immediately separated by centrifugation at 3500 rpm for 15 min. Serum 25(OH) D was measured with competitive chemiluminescent immunoassay in a calibrated Elecsys 2010 (Roche Diagnostics GmbH, Mannheim, Germany), with intra- and interassay coefficients of variation of 2.0–3.5% and 2.5–4.0%, respectively. The detection limit was 3 ng/ml. Other biochemical parameters (triglyceride, low and high-density lipoprotein, homocysteine (HCY), fasting blood glucose (FBG) and C-reactive protein (CRP)) were assessed using ROCHE COBAS C311 (Roche, Basel, Switzerland). Blood HbA1c was measured by HPLC (HLC-723 G7; Tosoh, Japan) with a normal range of 4–6%. For all measurements, levels that were not detectable were considered to have a value equal to the lower limit of detection of the assay. The 25(OH) D levels are therefore used to classify the vitamin D status into two groups as vitamin D deficiency (<20 ng/ml) and vitamin D sufficiency (≥20 ng/ml) [9]. Serum levels of parathyroid hormone (PTH) and calcium were available for a subgroup of 102 participants. PTH was measured with an automated analyzer using a sandwich principle by DPC Immulite 2000 (Diagnostic Products Corporation, CA, U.S.A.), and calcium was measured using the LX20 system that uses an indirect (or diluted) ISE methodology.

### Statistical analysis

The results were expressed as percentages for categorical variables and as medians (interquartile ranges, IQRs) for continuous variables. The Mann–Whitney U test and chi-square test were used to compare the two groups. The influence of 25(OH) D on poor functional outcomes and mortality was performed by binary logistic regression analysis, which allows adjustment for confounding factors (age, sex, BMI, infarct volume, NIHSS score, time from onset to blood collection, stroke syndrome, stroke etiology, pre-stroke and acute treatment, vascular risk factors and serum levels of Hs-CRP, FBG, HCY, HDL, LDL, and triglycerides). Results were expressed as adjusted odds ratio (OR) with the corresponding 95% confidence interval (CI). For a more detailed exploration of the 25(OH) D and functional outcomes, we also used multivariate analysis models to estimate adjusted OR and 95% CIs of poor functional outcomes for 25(OH) D quartiles (with highest 25(OH) D quartile as reference). In addition, the relationship between patients with vitamin D deficiency (compared with vitamin D sufficiency) and functional outcome (mortality) was also calculated. In a subgroup analyses (PTH was tested), the relationship between vitamin D deficiency and functional outcome (mortality) was calculated and adjusted for PTH and calcium. All statistical analysis was performed with SPSS for Windows, version 22.0 (SPSS Inc., Chicago, IL, U.S.A.). Statistical significance was defined as *P*<0.05.

## Results

### Patient characteristics

In the present study, 266 nondiabetic subjects with stroke were included and finished the 1-year follow-up (Table 1). Overall median age was 59 (IQR: 54–65) and 54.5% were male in the study population. The median (IQR) 25(OH) D was 18.4 (13.2–24.2) ng/ml; 149 out of the 266 patients were defined as vitamin D deficient (56%, 95% CI: 50–62%). There was a negative correlation between levels of 25(OH) D and NIHSS (*r* = –0.305, *P*<0.001). In patients for whom MRI data were available (*n*=148), there was also a negative correlation between levels of 25(OH) D and the infarct volume (*r* = –0.179, *P*=0.012).

**Table 1:** Baseline characteristics of nondiabetic stroke patients

Demographic characteristics	Patients
*n*	266
Male sex (%)	145 (54.5)
Age (years), median (IQR)	59 (54–65)
BMI (kg.m^−2^), median (IQR)	26.5 (24.9–28.6)
Stroke severity, median NIHSS score (IQR)	7 (3–14)
Vascular risk factors number (%)	
Hypertension	176 (75.9)
Atrial fibrillation	45 (19.4)
Coronary heart disease	65 (28.0)
Family history for stroke	51 (22.0)
Current cigarette smoking	55 (23.7)
Pre-stroke treatment, number (%)	
Anti-hypertensive treatment	142 (53.4)
Statins	62 (23.3)
Anticoagulants	41 (15.4)
Acute treatment, number (%)	59 (22.2)
TPA-T number (%)	41 (15.4)
Stroke etiology number (%)	
Small-vessel occlusive	51(19.2)
Large-vessel occlusive	58 (21.8)
Cardioembolic	102 (38.3)
Other	34 (12.8)
Unknown	21 (7.9)
Laboratory findings (IQR)	
Total cholesterol (mmol.l^−1^)	4.3 (3.4–5.3)
High-density lipoproteins (mmol.l^−1^)	1.3 (1.0–1.8)
FBG (mmol.l^−1^)	5.4 (5.1–5.8)
Hs-CRP (mg.dl^−1^)	0.64 (0.35–1.06)
tHCY (mmol.l^−1^)	19 (15–23)
25(OH) D (mmol.l^−1^)	18 (13–24)

Abbreviations: Hs-CRP, high CRP tHCY, total homocysteine; TPA-T, Tissue plasminogen activator treatment

### 25(OH) D and 1-year functional outcome

**Figure 1:**
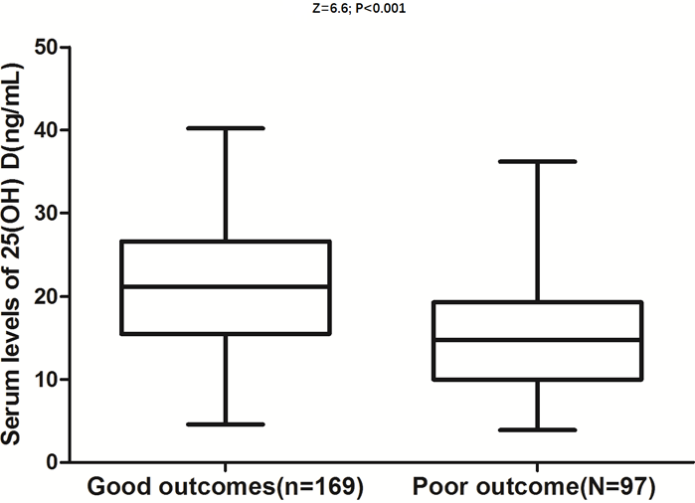
Distribution of 25(OH) D in stroke patients with poor functional outcomes and good functional outcomes Horizontal lines represent medians and IQRs. *P*-values refer to Mann–Whitney U tests for differences between groups. Poor functional outcome was defined as mRS in 3–6 point.

At follow-up, a poor functional outcome was found in 97 patients (37%; 95% CI: 31–42%) with a median mRS score of 4 (IQR: 3–6). The poor outcome distribution across the 25(OH) D quartiles ranged between 64% (first quartile) and 13% (fourth quartile) (Table 2). 25(OH) D in patients with good outcomes were significantly higher than those in patients with a poor outcome (21.2 (IQR: 15.5–26.6) compared with 14.8 (IQR: 10.0–19.3); Z = 6.6; *P*<0.0001; Figure 1). In univariate logistic regression analysis, we calculated the ORs of 25(OH) D as compared with the NIHSS score and other risk factors. With an unadjusted OR of 0.88 (95% CI: 0.85–0.92), 25(OH) D had a strong association with poor functional outcomes. After adjusting for all other significant outcome predictors, 25(OH) D remained an independent poor outcome predictor with an adjusted OR of 0.93 (95% CI: 0.88–0.96). After adjusting for other established risk factors, in multivariate models comparing the first (Q1) and second (Q2) quartiles against the fourth quartile (Q4) of the 25(OH) D, levels of 25(OH) D were associated with poor outcomes, and the adjusted risk of poor outcomes increased by 520% (OR = 6.2 (95% CI: 2.4–10.2), *P*<0.001) and 210% (3.1 (1.8–5.0), *P*<0.001), respectively (Table 2).

**Table 2:** ORs for poor outcomes according to 25(OH) D quarters at admission

25(OH) D quarters^1^	Outcomes, *n* (%)	Unadjusted OR (95% CI)^3^	Adjusted OR (95% CI) ^2^^,^^3^
Q1, *n*=67	43 (64)	11.6 (4.9–27.3)	6.2 (2.4–10.2)
Q2, *n*=66	27 (41)	4.5 (1.9–10.5)	3.1 (1.8–5.0)
Q3, *n*=66	18 (27)	2.4 (1.0–5.9)	1.6 (0.9–3.1)
Q4, *n*=67	9 (13)	Reference	Reference

1 Serum levels of 25(OH) D in Quartile 1 (<13.2 ng/ml), Quartile 2 (13.2–18.4 ng/ml), Quartile 3 (18.5–24.2 ng/ml), and Quartile 4 (>24.2 ng/ml).

2 Adjusted for age, sex, infarct volume, BMI, NIHSS score, season of samples included, time from onset to blood collection, stroke syndrome, stroke etiology, treatment, vascular risk factors and blood levels of cholesterol, HDL, homocysteine, FBG, and CRP.

3 *P*-value for the trend <0.001.

### 25(OH) D and 1-year mortality

**Figure 2:**
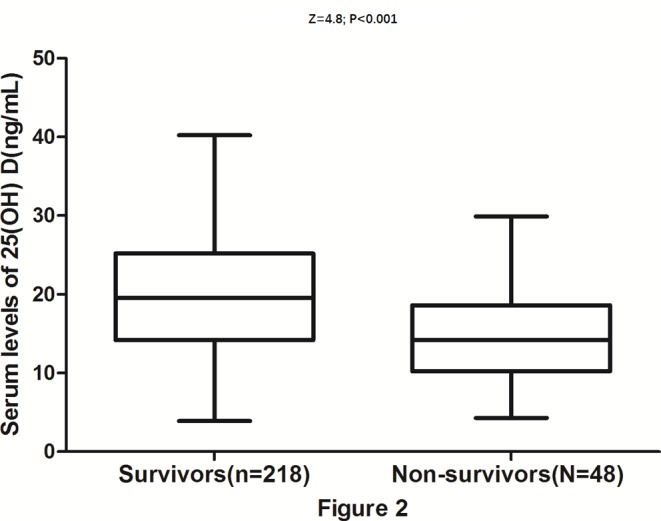
Distribution of 25(OH) D in survivors and nonsurvivors of stroke Horizontal lines represent medians and IQRs. *P*-values refer to Mann–Whitney U tests for differences between groups.

After 1 year, 48 patients had died, thus the mortality rate was 19% (95% CI: 13–23%). Serum 25(OH) D levels in patients who survived were significantly greater as compared with patients who died (19.9 (IQR: 14.3–25.2) compared with 9.8 (IQR: 14.2–18.2) ng/ml; Z = 4.8; *P*<0.001), Figure 2. The distribution of mortality across the 25(OH) D quartiles ranged between 33% (first quartile) and 6% (fourth quartile) (Table 3). After adjustment for other parameters, 25(OH) D levels remained an independent predictor for mortality with an OR of 0.95 (95% CI: 0.91–0.98; *P*=0.001). After adjusting for other established risk factors, in multivariate models comparing the first (Q1) and second (Q2) quartiles against the fourth quartile (Q4) of the 25(OH) D, levels of 25(OH) D were associated with poor outcomes, and the adjusted risk of poor outcomes increased by 350% (OR = 4.5 (95% CI: 2.0–9.1), *P*<0.001) and 170% (2.7 (1.6–4.9), *P*=0.001), respectively (Table 3).

**Table 3:** ORs for mortality according to 25(OH) D quarters at admission

25(OH) D quarters^1^	Mortality, *n* (%)	Unadjusted OR (95% CI)^3^	Adjusted OR (95% CI)^2^^,^^3^
Q1, *n*=67	22 (33)	7.7 (2.5–23.9)	4.5 (2.0–9.1)
Q2, *n*=66	14 (21)	4.2 (1.3–13.7)	2.7 (1.6–4.9)
Q3, *n*=66	8 (12)	2.2 (0.6–7.6)	1.4 (0.7–6.9)
Q4, *n*=67	4 (6)	Reference	Reference

1 Serum levels of 25(OH) D in Quartile 1 (<13.2 ng/ml), Quartile 2 (13.2–18.4 ng/ml), Quartile 3 (18.5–24.2 ng/ml), and Quartile 4 (>24.2 ng/ml).

2 Adjusted for age, sex, infarct volume, BMI, NIHSS score, season of samples included, time from onset to blood collection, stroke syndrome, stroke etiology, treatment, vascular risk factors and blood levels of cholesterol, HDL, homocysteine, FBG, and CRP.

3 *P*-value for the trend <0.001.

### Vitamin D deficiency and stroke outcomes

In those 149 patients with vitamin D deficiency, 75 patients were defined as poor functional outcomes, giving a prevalence rate of 50% (95% CI: 42–58%). In contrast, 19% (22/117; 95% CI: 12–26%) of the vitamin D sufficiency acknowledged poor functional outcomes. The difference between groups was statistically significant (OR: 4.2; 95% CI: 2.4–7.3; *P*<0.001). Furthermore, in multivariate analysis models, for vitamin D deficiency, the adjusted risk of poor functional outcomes was increased by 220% (OR: 3.2; 95% CI: 1.7–4.2, *P*<0.001). Similarly, 40 and 8 patients died in vitamin D deficiency and vitamin D sufficiency groups, respectively. The difference between groups was statistically significant (OR: 5.0; 95% CI: 2.2–11.2; *P*<0.001). Again, in multivariate analysis models, for vitamin D deficiency, the adjusted risk of mortality increased by 290% (OR: 3.9; 95% CI: 2.1–5.8, *P*<0.001).

### A subgroup analysis

In those 102 patients whose PTH and calcium had been tested, 36 patients had been defined as poor functional outcomes, while 20 patients died. In multivariate analysis models, adjusted for age, sex, infarct volume, BMI, NIHSS score, season of samples included, time from onset to blood collection, stroke syndrome, stroke etiology, treatment, vascular risk factors and blood levels of cholesterol, HDL, HCY, FBG, CRP, PTH, and calcium, vitamin D deficiency was associated with poor functional outcomes, and the risk increased by 200% (OR: 3.0; 95% CI: 1.6–4.1, *P*<0.001). Furthermore, vitamin D deficiency was also associated with mortality, and the risk increased by 250% (OR: 3.5; 95% CI: 1.5–5.1, *P*<0.001).

## Discussion

In this prospective, population-based cohort study of nondiabetic individuals, we report that vitamin D deficiency estimated using 25(OH) D is associated with a 3.2-fold increased risk of poor functional outcome events. Adjustment for established cardiovascular risk factors, including glucose level, age, and NIHSS score, did not attenuate this association. Furthermore, for vitamin D deficiency, the adjusted risk of mortality increased by 290% (OR: 3.9; 95% CI: 2.1–5.8, *P*<0.001). To our knowledge, the present study is a novel finding and has not been previously described. It is imperative to emphasize targetted lifestyle intervention and more frequent medical interventions for nondiabetic stroke patients, especially for the patients with vitamin D deficiency.

Consistent with our results, several observational studies have reported a protective effect of vitamin D on functional outcomes and mortality of ischemic stroke [6,10,17–19]. It has been suggested that vitamin D has neuroprotective properties [20] and vitamin D supplementation could be beneficial to reduce the volume of cerebral infarct in animal models of stroke [21]. Interestingly, another study reported that low 25(OH) D was associated with an increased risk of cardiovascular morbidity and mortality in people with type 2 diabetes independent of PTH [22].

Hyperglycemia is common amongst acute stroke patients because of stress response or previous diabetes. Diabetes is regarded as an independent risk factor for ischemic stroke prognosis [23]. China National Stroke Registry showed that 28% ischemic stroke patients had diabetes [24]. In our study of nondiabetic stroke individuals, vitamin D deficiency is associated with increased risk of poor functional outcome events. Similarly, a previous study suggested that serum 25(OH) D deficiencies may be merely an independent risk factor of 1-year poor prognosis in ischemic stroke patients without hyperglycemia [25]. Thus, we confirm that effect of 25(OH) D on ischemic stroke prognosis is not modified by blood glucose concentrations.

Whether vitamin D supplementation at adequate doses can improve outcomes in those patients need further investigation. However, in the present study, the observational study does not allow advancing any cause and effect relationships. Lindqvist et al. [26] found that the longer life expectancy amongst women with active sun exposure habits was related to a decrease in CVD and noncancer/non-CVD mortality. However, an inverse association between outdoor recreational activity (ORA) and CVD mortality was observed independent of 25(OH) D [27]. Another study suggested that UV radiation (UVR) exposure might not be beneficial for longevity [28].

Some possible biologic mechanisms might explain the protective mechanisms of vitamin D3 in stroke outcomes and reference. First, inflammation has a significant role in the pathogenesis of ischemic stroke. Low 25(OH) D concentrations are known to influence macrophage and lymphocyte activity in atherosclerotic plaques and to promote chronic inflammation in the artery wall [29]. Alfieri et al. [6] suggested that the important role of vitamin D in the anti-inflammatory response and pathophysiology of this ischemic event. Second, vitamin D deficiency has been associated with morphologic brain changes and motor impairments in animal models [10]. Additionally, some clinical studies have indicated that vitamin D deficiency was associated with accelerated bone resorption and reduced bone mineral density in stroke patients [30]. Third, vitamin D deficiency might contribute to pro-atherosclerotic changes of vascular smooth muscle cells, endothelial dysfunction, and increased macrophage to foam cell formation [30]. Witham et al. [31] found that high dose oral vitamin D supplementation produced short-term improvement in endothelial function in stroke patients with well-controlled baseline blood pressure (BP). Fourth, a U-shaped relationship was found between baseline systolic BP and both early death and late death or dependency [32]. Furthermore, improved serum 25(OH)D concentrations in hypertensive individuals who were vitamin D insufficient were associated with improved control of systolic and diastolic BP [33]. Another study [34] suggested that monthly, high‐dose, 1‐year vitamin D supplementation lowered central BP parameters amongst adults with vitamin D deficiency but not in the total sample. Opländer et al. [35] found that UVA irradiation of human skin caused a significant drop in BP even at moderate UVA doses. However, another study concluded that although 25(OH)D concentration was inversely associated with SBP, 25(OH)D it did not explain the association of greater sunlight exposure with lower BP [36]. Finally, the observation of reduced mortality risk with 1,25(OH)2 D supplements amongst patients with renal failure [37] and general population [38] supports a possible CVD protective role of vitamin D.

The present study has several strengths that deserve mention. To avoid the confounding influence of glycemia, patients present with acute hyperglycemia were excluded from our study. Further, we chose a different strategy using the four quartiles, a more complete understanding of the effect of 25(OH) D on the distribution of stroke outcomes can be obtained. The present study also has some limitations. First, the relatively small sample size (*n*=266) may limit the generalization of the results of the present study. In addition, potential confounding factors, including serum PTH and calcium might influence the relationship between 25(OH) D and stroke outcomes. In the present study, we only adjusted PTH and calcium in a subgroup analyses (*n*=102). However, the PTH and calcium could not change the association between vitamin D deficiency and functional outcome events. Vitamin D and PTH might influence stroke outcomes through divergent pathway. Second, fasting blood samples used to determine 25(OH) D were obtained during the first 24 h after stroke onset and only once. Without serial measurement of the circulating 25(OH)D, the present study yielded no data regarding when and how long this biomarker was reduced in these patients. Additionally, it should be investigated whether serial 25(OH) D testing further improves the risk stratification of stroke patients. Interestingly, most of our patients in the present study had vitamin D deficiency (56%). However, vitamin D deficiency is common (75.2%) in Chinese population [39] and our cohort is not atypical. A previous study in Chinese stroke patients found that 78.2% patients suffered from vitamin D deficiency [10]. Third, the observational study does not allow advancing any cause and effect relationships. However, a previous study suggested that vitamin D in combination with hypothermia supported functional recovery in both sexes of neonatal rats with severe hypoxic ischemic encephalopathy [40]. In addition, we did not collect data on sun exposure, dietary intake of vitamin D, and outdoor physical activity, so we could not determine the association of those factors with serum 25(OH) D levels and outcomes of Chinese patients with acute ischemic stroke. Fourth, there is evidence that vitamin D may favorably influence stroke outcomes through multiple pathways, including hypertension, insulin resistance and secretion, and chronic inflammation. The inclusion of those factors in the models could possibly lead to overadjustment, which tends to attenuate the associations. Last, a significant section of the population is either pre-diabetic or living with undiagnosed diabetes, which may not be evident from a single blood measurement. Thus, this may serve as a confounding factor in the present study, and some patients with undiagnosed diabetes might be included in the study.

## Conclusion

Vitamin D deficiency is associated with an increased risk of poor functional outcome events in Chinese nondiabetic stroke individuals. However, it is currently unknown whether vitamin D supplementation at adequate doses can improve prognosis in those patients. Additional randomized controlled trials are therefore urgently needed.

## Abbreviations

BMI: body mass index
BP: blood pressure
CI: confidence interval
CRP: C-reactive protein
CVD: cardiovascular disease
FBG: fasting blood glucose
HbA1c: glycated hemoglobin
HCY: homocysteine
HDL: high-density lipoprotein
IQR: interquartile range
IV: Intravenous
LDL: Low-density Lipoprotein
mRS: modified Rankin scale
NIHSS: National Institutes of Health Stroke Scale
OR: odds ratio
PTH: parathyroid hormone
SBP: Systolic blood pressure
25(OH) D: 25-hydroxyvitamin D
